# Investigation of Fracturing Fluid Flowback in Hydraulically Fractured Formations Based on Microscopic Visualization Experiments

**DOI:** 10.3390/polym15061560

**Published:** 2023-03-21

**Authors:** Guodong Zou, Bin Pan, Weiyao Zhu, Yuwei Liu, Shou Ma, Mingming Liu

**Affiliations:** 1 School of Civil and Resource Engineering, University of Science and Technology Beijing, Beijing 100083, China; 2 SinoFTS Petroleum Services Ltd., Beijing 100083, China

**Keywords:** polyacrylamide, shale gas, hydraulic fracture, fracturing fluid flowback, microscopic model

## Abstract

Fracturing fluids are widely applied in the hydraulic fracturing of shale gas reservoirs, but the fracturing fluid flowback efficiency is typically less than 50%, severely limiting the shale gas recovery. Additionally, the mechanism and main influencing factors of fracturing fluid flowback are unclear. In this study, microscopic experiments are conducted to simulate the fracturing fluid flowback progress in shale gas reservoirs. The mechanism and factors affecting fracturing fluid flowback/retention in the fracture zone were analyzed and clarified. Results show that the ultimate flowback efficiency of fracturing fluid is positively correlated with the fracturing fluid concentration and the gas driving pressure difference. There are four kinds of mechanisms responsible for fracturing fluid retention in the pore network: viscous resistance, the Jamin effect, the gas blockage effect and the dead end of the pore. Additionally, the ultimate flowback efficiency of the fracturing fluid increases linearly with increasing capillary number. These insights will advance the fundamental understanding of fracturing fluid flowback in shale gas reservoirs and provide useful guidance for shale gas reservoirs development.

## 1. Introduction

Shale gas, an unconventional natural gas with huge potential and low carbon emissions, is currently one of the key energy sources being developed and exploited around the world [1]. Nowadays, four countries—China, the United States, Argentina and Canada—have achieved commercial development of shale gas [2,3]. Apart from them, Australia, Mexico, South Africa, and the United Kingdom are also actively promoting it [4,5]. Hydraulic fracturing is a critical technology in the shale gas development. The recovery of shale gas is closely related to the effectiveness of hydraulic fracturing, and the flowback efficiency of the fracturing fluid is an important quantitative indicator. In the hydraulic fracturing process, more than 10,000 m^3^ fracturing fluids are pumped into the shale reservoir, but the flowback efficiency is typically less than 50% and a large amount of fracturing fluid remains in the shale reservoir, severely limiting the shale gas recovery [6,7]. Therefore, the understanding of factors that influence the fracturing fluid flowback in shale gas reservoirs is urgently required to accelerate the development.

Polymers are frequently employed in water-based fracturing fluids with the primary purposes of minimizing turbulence-induced friction and enhancing the fracturing fluid’s ability to carry proppant particles. This is achieved through the elevation of the fracturing fluid’s viscosity, transforming it into a non-Newtonian fluid [8]. The commonly used polymers added to water-based fracturing fluids are shown in Table 1. In addition to water-based fracturing fluids, silicate-based fracturing fluids are also frequently used for hydraulic fracturing of unconventional gas reservoirs. The main components in common silicate based fracturing fluids are sodium silicate, silicate gel and aluminum silicate. The differences between water-based and silicate fracturing fluids are shown in Table 2.

Based on the flow mechanism in the shale reservoir after hydraulic fracturing, scholars have applied different models to simulate the flow of fluids during the flowback process. From the early single-phase flow [9], to two-phase flow models [10], and more recently to simulation studies that have taken into account various flowback mechanisms [11]. In addition to mathematical modelling and numerical simulation, another research tool is to simulate the fracturing fluid invasion and flowback process through the indoor experiments with core plugs. The experiments include dynamic and static imbibition and aim to investigate factors such as fracturing fluid properties, surface area of artificial fracture networks, shale hydration capacity and well shut-in time that affect flowback efficiency [12]. The indoor experiments with core plugs simulate the fracturing fluid invasion/ flowback process and the recovery of oil and gas on a macroscopic scale, but do not support studies of fluid transport on a microscopic pore scale. As an emerging method, microscopic models allow researchers to study the flow of fluids quantitatively and visually in microscopic pores. Scholars have used microscopic models to study the progress of fracturing fluid flows in recent years [13,14].

When gravity is negligible, the capillary force is an important factor influencing the transport of immiscible two-phase fluids at the pore level [15]. The capillary force is quantified by an important dimensionless number—the capillary number. Scholars have investigated the relationship between residual oil saturation and capillary number, i.e., study the capillary desaturation curve (CDC). However, the relationship between fracturing fluid flowback efficiency and the capillary number for gas-fracturing fluid two-phase, which are also immiscible two-phase fluids, has rarely been studied [16,17].

In order to investigate the factors influencing the fracture fluid flowback efficiency in shale gas reservoirs, microscopic models are applied in this study. In this field of application, a microscopic model is fabricated with a custom designed porous network etched on its surface that is used to inject desired solutions at certain operating conditions such as invasion amounts, flowback rates and injection pressures [14]. Experiments were carried out on the model with gas driving fracturing fluids, and then the results were used to analyze the flowback behavior of fracturing fluids in shale gas reservoirs. The mechanism of the fracturing fluid flowback is investigated from the microscopic perspectives of capillary force, viscous force and capillary number. The results obtained provide a better understanding of fracturing fluid flowback after hydraulic fracturing in shale gas reservoirs, which can provide theoretical guidance for shale gas production.

## 2. Materials and Methods

### 2.1. Experimental Materials and Fluid Properties

**Figure 1:**
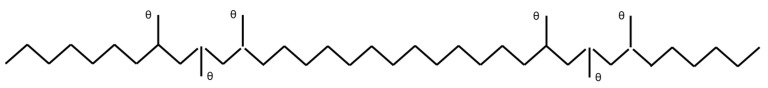
The molecular structure characteristics of polyacrylamide polymer.

**Figure 2:**
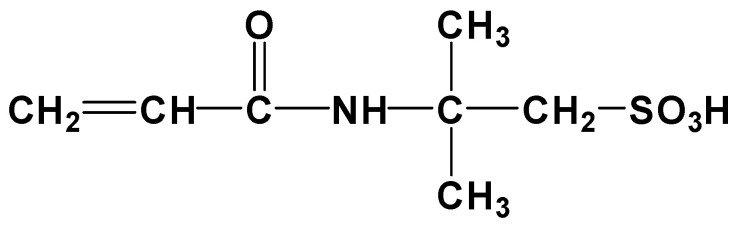
The structural characteristics of AMPS-AM.

A new polyacrylamide polymer fracturing fluid provided by Dingshan gas field was used in this study. PAM with a molecular weight of 26.8 million Daltons, a solid content of 89.0% and a hydrolysis degree of 19.7%. From a molecular design perspective, incorporating micro-block molecule models in polymerization maintains a linear molecular structure and improves solubility. The charge in the middle of each block is minimally affected by the environment, maintaining a certain degree of Coulomb repulsion between them, which results in a relatively large hydrodynamic radius for the polymer, thus maintaining high molecular weight and solution viscosity. The molecular structure characteristics are shown in Figure 1. By introducing a salt-insensitive sulfonic acid AMPS-AM group between molecules, the impact of divalent ions on carboxylic acid groups is reduced, decreasing the sensitivity of drag reducers to produced water and temperature and increasing their tolerance to temperature, salt, and long-term stability. The structural characteristics are shown in Figure 2.

**Figure 3:**
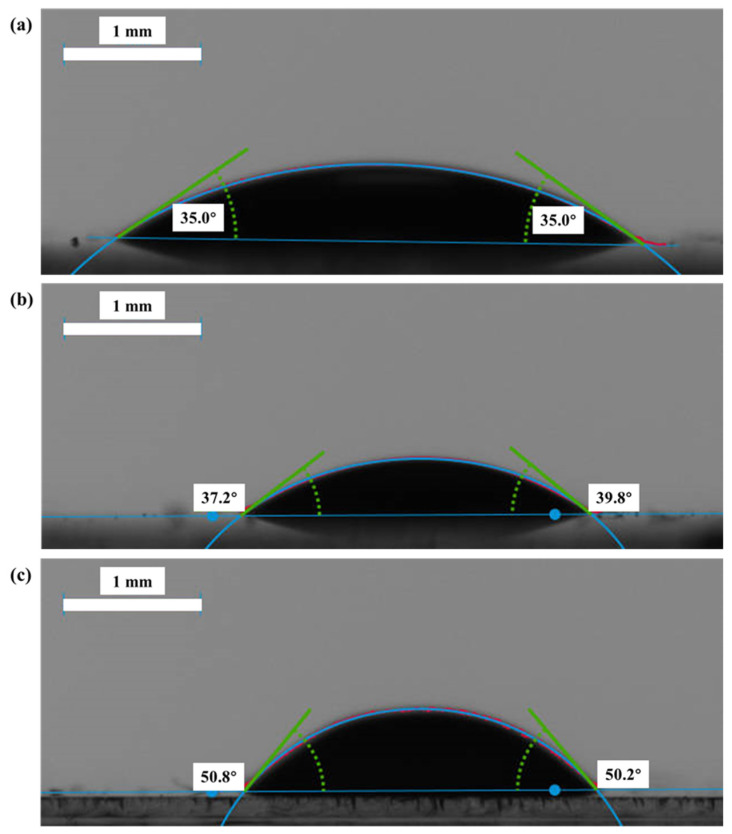
The contact angles of fracturing fluid-nitrogen and glass surface: (**a**) 0.1 wt% fracturing fluid; (**b**) 0.8 wt% fracturing fluid; (**c**) 1.2 wt% fracturing fluid.

Experimental fracturing fluids with concentrations of 0.1 wt%, 0.8 wt% and 1.2 wt% were prepared using DI water, with viscosities of 7.1 mPa·s, 62.7 mPa·s and 96.1 mPa·s, respectively (the shear rate for viscosity testing was 511 s^−1^, and the temperature was 20 °C). High-purity nitrogen with a concentration of 99.999% and a viscosity of 0.01757 mPa·s (20 °C, 101 KPa) was used as the experimental gas. The interfacial tension (IFT) between nitrogen and fracturing fluid was measured by a tensiometer at 20 °C. The contact angles of fracturing fluid-nitrogen and glass surface were measured using KRUSS DSA100S (within 60 ± 10 s after the liquid droplet transfer, Hamburg, Germany), as shown in Figure 3. The properties of the fluids are listed in Table 3.

### 2.2. Microscopic Model Fabrication

Fluid flow in a reservoir is influenced by a number of factors, including gravity, capillary pressure, pore throat structure, viscous force and driving force [18,19]. The current microscopic models include idealized grid models and complex structural models with tortuousness [20]. Despite omitting consideration of the pore throat structure, the idealized model has the advantage of allowing direct observation of the changes in three-phase contact surface after wetting modification [21]. Nevertheless, when subjected to high driving forces, it is relatively effortless for the liquid phase in the model to be totally expelled, making simulation of the additional resistance effects of tortuous porous media near-impossible. Alternatively, a complex structure model was designed based on the real pore throat of cores, which includes tortuousness structure [22].

**Figure 4:**
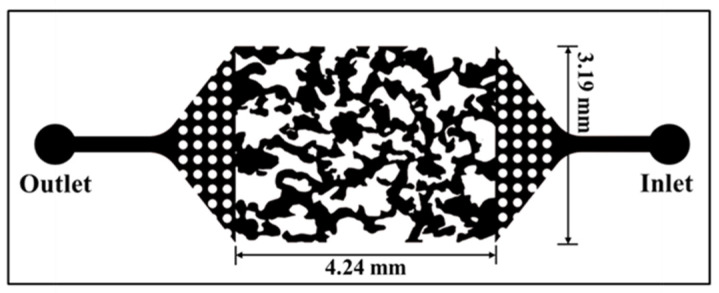
Schematic diagram of the microscopic model.

The experiment was centered around the microscopic model (Figure 4) with a pore structure that was delineated following the cross-sectional image of a fractured shale core plug and was etched onto a flat glass plate. Another glass plate was placed over this plate in order to create a pore space enclosed within it. In the cover plate, there exists an inlet hole and an outlet hole, which facilitate the flow of fluids through the pore network. The external size, pore volume and pore depth were 635 mm × 318 mm, 7 μL and 25–35 μm, respectively. The size of rectangular area in the center was 4.24 mm × 3.19 mm.

In the microscopic model, the role of the dotted area on both sides is, firstly, to simulate the high porosity area of the main fracture after hydraulic fracturing, and secondly, to obtain a uniform flow rate and a more stable injection rate [23]. The rectangular area in the center, patterned from real core, simulates the branch fractures and natural fracture areas. The fracturing fluid was injected from the outlet hole until the model was completely saturated with fracturing fluid, and then injects nitrogen from the inlet hole, simulating exactly the process of fracturing fluid flowback from the branch and natural fractures to the main fracture/wellbore area. This study focuses on fracturing fluid concentration and driving pressure, two factors that affect fracturing fluid flowback, and this microscopic model can effectively complete the experiment.

### 2.3. Experimental Setup and Procedure

**Figure 5:**
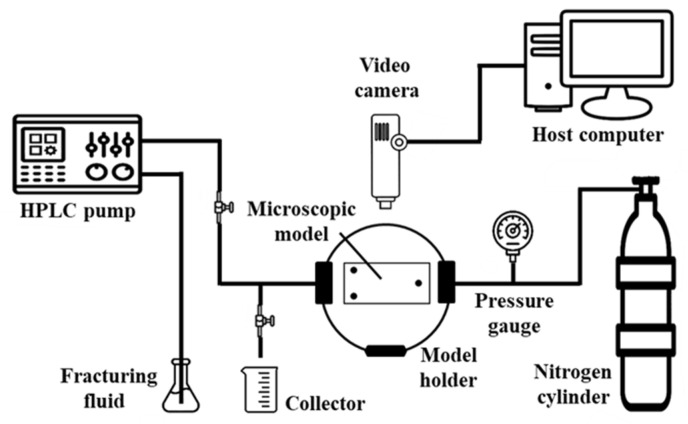
Schematic diagram of experiment.

The schematic of experimental setup is shown in Figure 5. After loading the microscopic model into the model holder, the HPLC pump and the nitrogen cylinder were connected to the outlet and inlet of the model holder by tubing lines, respectively. The processes of the fracturing fluid invasion and flowback were recorded by the video camera. The experiment was conducted at 20 °C.

The specific experimental procedure was as follows:

1. Evacuate the microscopic model for 1 h to prevent other gases from entering the model [24];
2. The HPLC pump was used to inject fracturing fluid into the microscopic model at a rate of 5 μL/min, and the pump was stopped when the model was completely saturated;
3. The nitrogen gas was injected into the microscopic model under different pressure differences (from 0.1 to 0.5 MPa), then observe and record the retention and distribution of the liquid phase in the microscopic model at each pressure, and close the nitrogen cylinder when there was no longer any significant change;
4. Clean the model and repeat steps 1–3 after changing the fracturing fluid concentration and adjusting the nitrogen injection pressure.

All experiments are conducted on the identical microscopic model, with every individual experiment preceded by a cleaning sequence that includes multiple rounds of DI water flushing, vacuuming, heating, and drying. Then, place the model under the microscope to ensure that there is no residual liquid in the pore network.

### 2.4. Image Processing

In order to quantify the fracturing fluid flowback and to further compare the flowback efficiencies of the different experimental groups, the experimental images were processed using ImageJ software. The experimental image was first sharpened, then the background was removed and the image was converted to a grey-scale binary image. In the binary image, the fracturing fluid is highlighted by the difference in gray-scale values between the fracturing fluid and the nitrogen [25,26]. The fracturing fluid saturation can then be calculated from the percentage of black areas in the pore space. The fracturing fluid flowback efficiency (*FB*%) can be calculated as shown in Equation (1):

(1) $$FB\%=1-\frac{S_{f}}{S_{f0}},$$

where $S_{f}$ represents the fracturing fluid saturation in the microscopic model after gas driving; $S_{f0}$ represents the fracturing fluid saturation at initial time.

## 3. Results

The results of the fracturing fluid flowback experiments are shown in Table 4.

**Figure 6:**
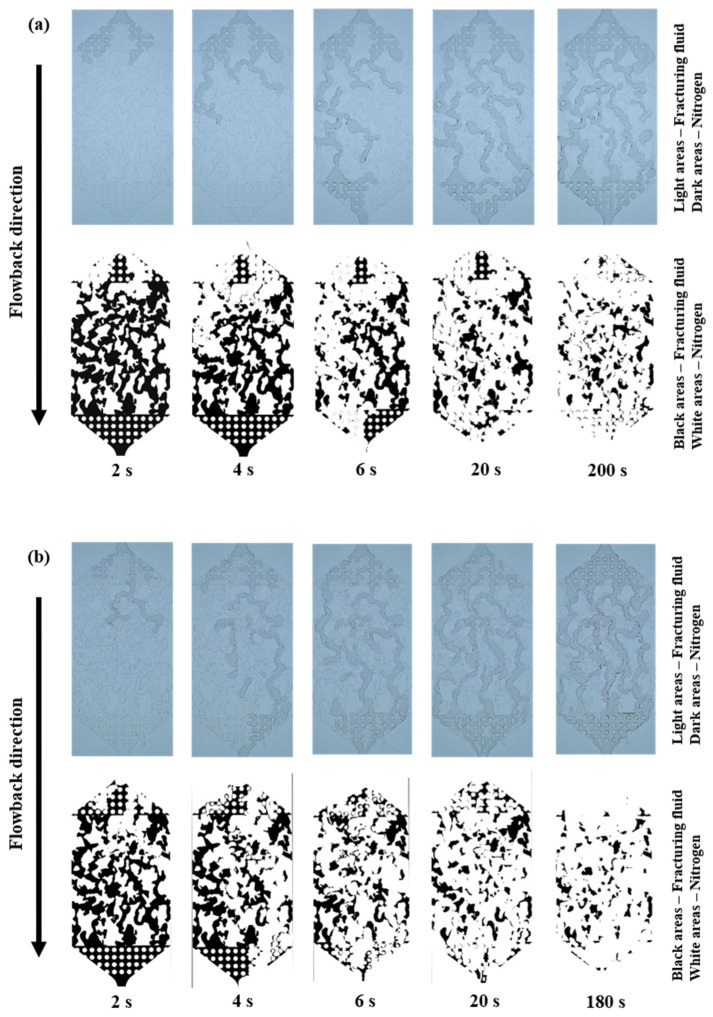

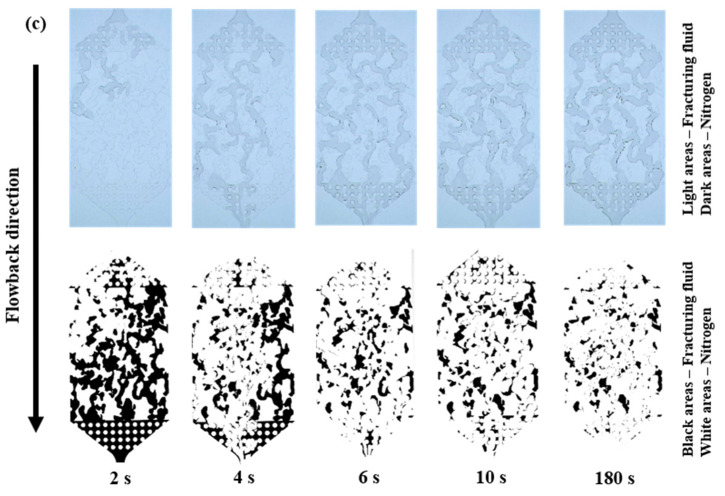
The fracturing fluid (0.1 wt%) flowback process images and binary images (**a**) 0.1 MPa gas drive, (**b**) 0.2 MPa gas drive, (**c**) 0.3 MPa gas drive.

The process of fracturing fluid flowback from the branch fractures to the main fracture was shown in Videos S1–S3 and Figure 6. The concentration of the fracturing fluid was 0.1 wt% and the gas driving pressure differences of the three groups were 0.1, 0.2 and 0.3 MPa, respectively. During the early stage of flowback (0–6 s), the gas quickly broke through and advanced along the fractures of minimal resistance to reach the main fracture zone of greater porosity and permeability. In the middle stage (6–20 s), other gases that had not completed their breakthrough, flowed into the fractures of minimal resistance, expanding the gas flow area. Concurrently, the main fracture zone experienced a rapid influx of the fracturing fluid, resulting in a noteworthy enhancement in flowback efficiency. In the later stage (20–200 s), gas flow channels had been formed and most of the fracturing fluid had been driven out, and the residual was mainly concentrated in the dead end and smaller pore fractures. Over time, the residual fracturing fluid did not change much in terms of content.

**Figure 7:**
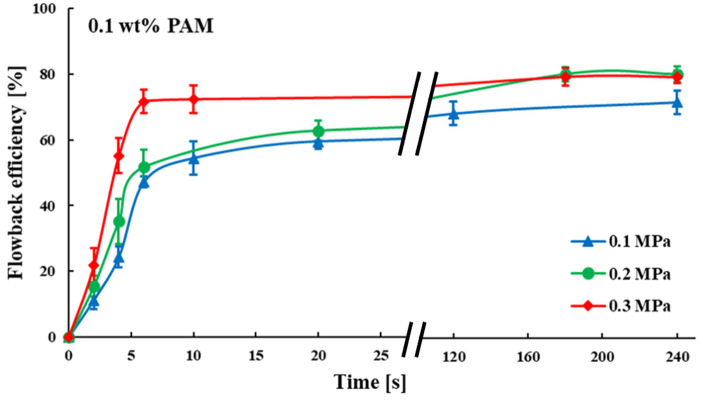
Relationship between time and flowback efficiency of fracturing fluid (0.1 wt%).

Figure 7 shows the relationship between the fracturing fluid flowback efficiency and time, under three sets of gas driving pressure differences at 0.1 wt% fracturing fluid concentration. It was clear that there was a rapid increase in flowback efficiency in the early stages, then a plateau. This trend was observed in all three groups of experiments. The ultimate fracture fluid flowback efficiencies for each group were approximately the same under different gas driving pressure differences (0.1, 0.2 and 0.3 MPa) with little difference (71.417%, 79.992% and 79.109%).

**Figure 8:**
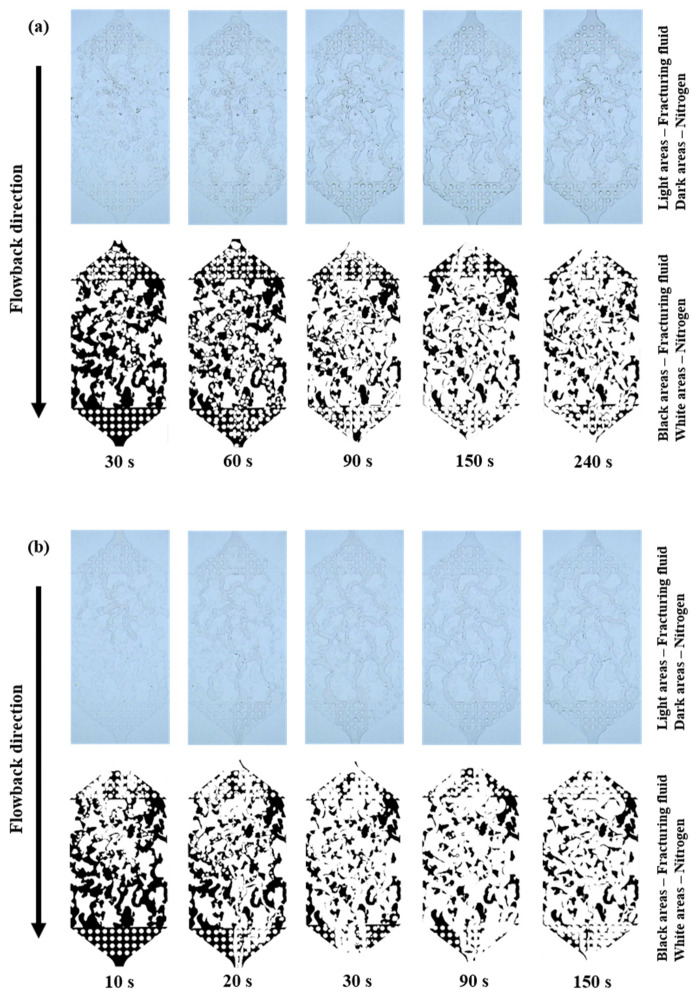

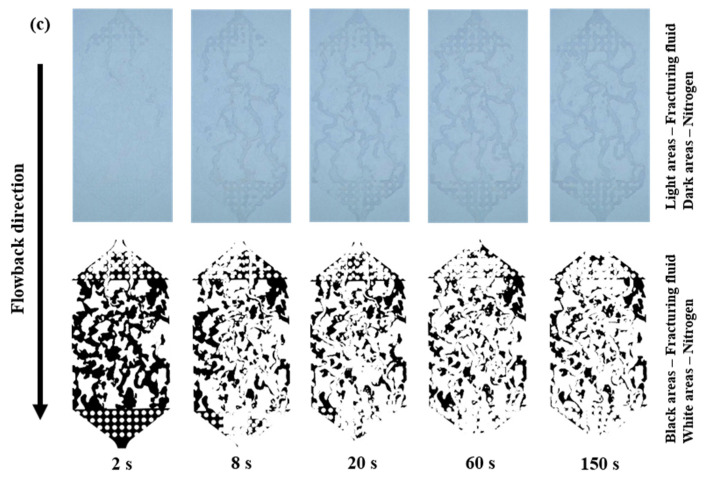
The fracturing fluid (0.8 wt%) flowback process images and binary images (**a**) 0.1 MPa gas drive, (**b**) 0.2 MPa gas drive, (**c**) 0.3 MPa gas drive.

**Figure 9:**
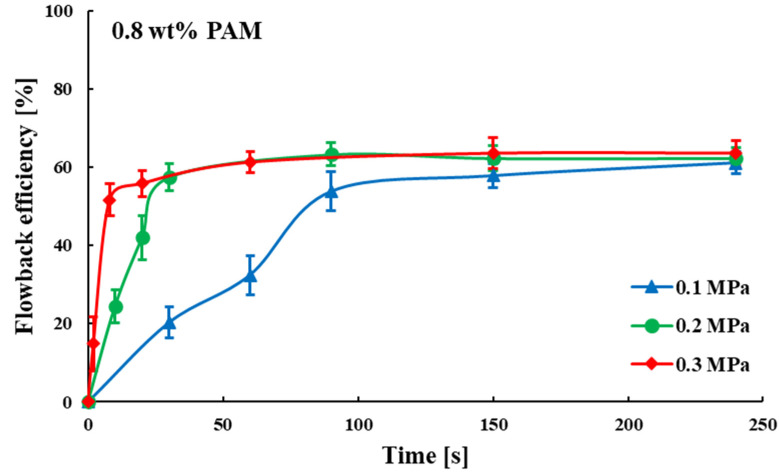
Relationship between time and flowback efficiency of fracturing fluid (0.8 wt%).

To assess the impact of fracturing fluid concentration on the fluid flowback behavior, the experimental procedure was conducted once more with a heightened fracturing fluid concentration of 0.8 wt%. Additionally, the gas driving pressure differences remained at 0.1, 0.2 and 0.3 MPa for the three groups. The process of fracturing fluid flowback was shown in Videos S4–S6 and Figure 8. During the early stage of fracturing fluid flowback in group (a) (0.1 MPa gas drive), the gas was not in a continuous phase but dispersed into small bubbles that flowed into the fracturing fluid in the fractures. Again, the gas advanced along the fractures of minimal resistance to reach the main fracture zone of greater porosity and permeability. However, this breakthrough process took longer than the 0.1 wt% fracturing fluid experimental group. In the middle stage, after breakthrough, the gas bubbles quickly turned into a continuous phase, and other gases that had not broken through, some flowed into the fractures of minimal resistance, the other part remained bubbles and stayed in the dead end and smaller pore fractures. There was a significant reduction in fracturing fluid saturation and a rapid increase in flowback efficiency during these two stages. Groups (b) and (c) had higher gas driving pressure differences and correspondingly shorter early stage of fracturing fluid flowback, specifically (c) < (b) < (a). In the later stage, as in the 0.1 wt% fracturing fluid experimental group, the residual fracturing fluid saturation remained constant after the gas flow channels had formed. Additionally, as shown in Figure 9, the ultimate fracture fluid flowback efficiencies for each group remained approximately the same with little difference (61.157%, 62.313%, 63.568%) under different gas driving pressure differences (0.1, 0.2, 0.3 MPa).

**Figure 10:**
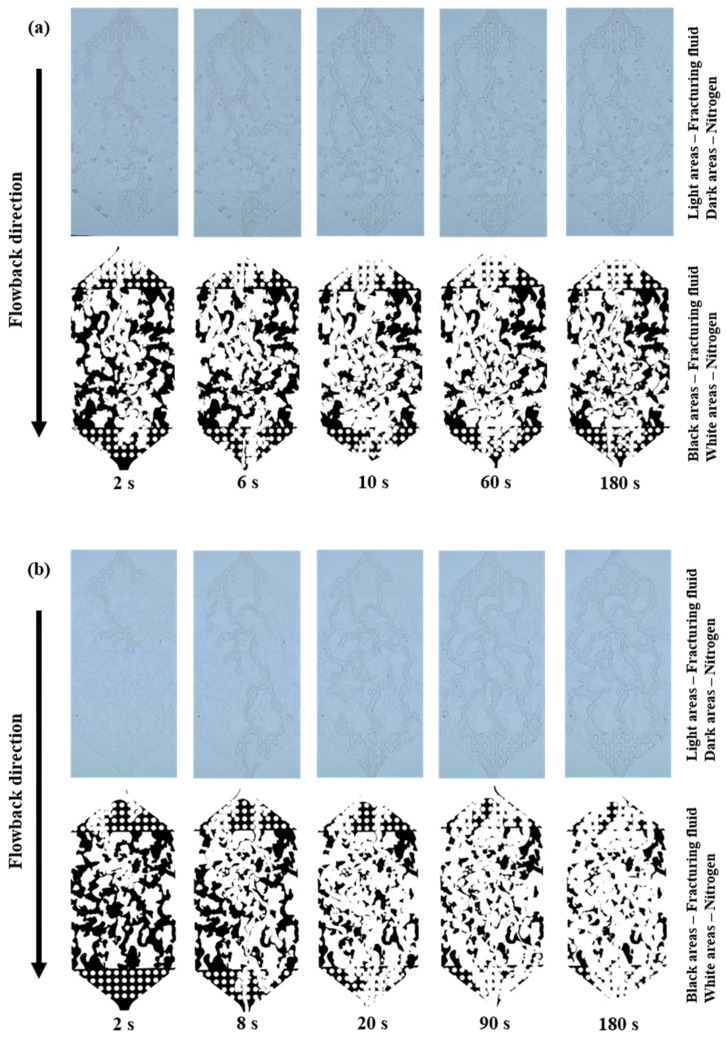

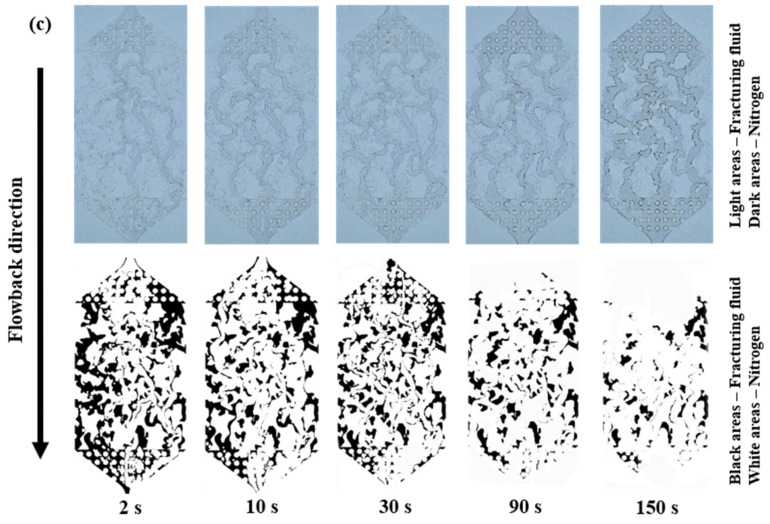
The fracturing fluid (1.2 wt%) flowback process images and binary images (**a**) 0.3 MPa gas drive, (**b**) 0.4 MPa gas drive, (**c**) 0.5 MPa gas drive.

**Figure 11:**
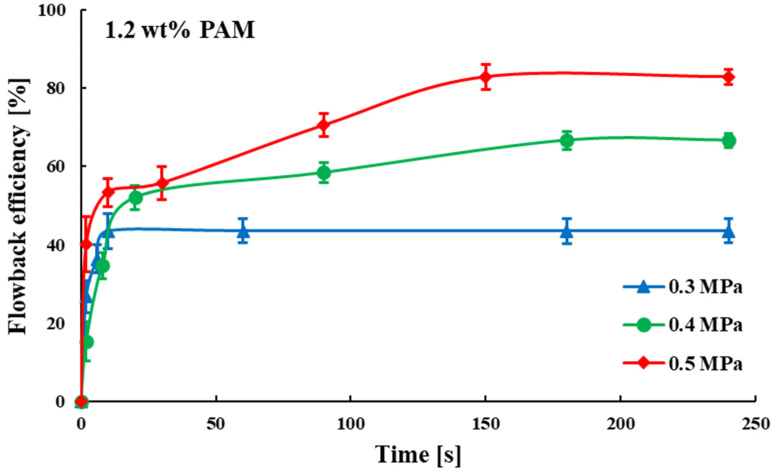
Relationship between time and flowback efficiency of fracturing fluid (1.2 wt%).

Compared to the first two sets of experiments, the third set further increased the fracturing fluid concentration (1.2 wt%), while adjusting the gas driving pressure differences to 0.3, 0.4 and 0.5 MPa. As shown in Videos S7–S9 and Figure 10, during the early stage of flowback in all three experimental groups, the gas broke through rapidly and flowed as a continuous phase in the dominant channel. Meanwhile, there were still some bubbles in the fracturing fluid. In group (a) (0.3 MPa gas drive), the fracturing fluid flowback efficiency increased rapidly and then plateaued: once the dominant channel was formed, most of the subsequent gas would flow through it. In groups (b) and (c), fracturing fluid flowback efficiency displayed a prompt escalation in its early stage, ensuing in gradual increases during the mid stage, culminating in a plateau: after the gas breakthrough, some gas continued to expand the flow area and connected up to other fractures. In contrast to the first two sets, the 1.2 wt% fracturing fluid experimental set, as shown in Figure 11, had greater difference in flowback efficiencies (61.157%, 62.313%, 63.568%) under different gas driving pressure differences (0.3, 0.4, 0.5 MPa).

## 4. Discussion

### 4.1. Retention Mechanism of Fracturing Fluid in the Micromodel

**Figure 12:**
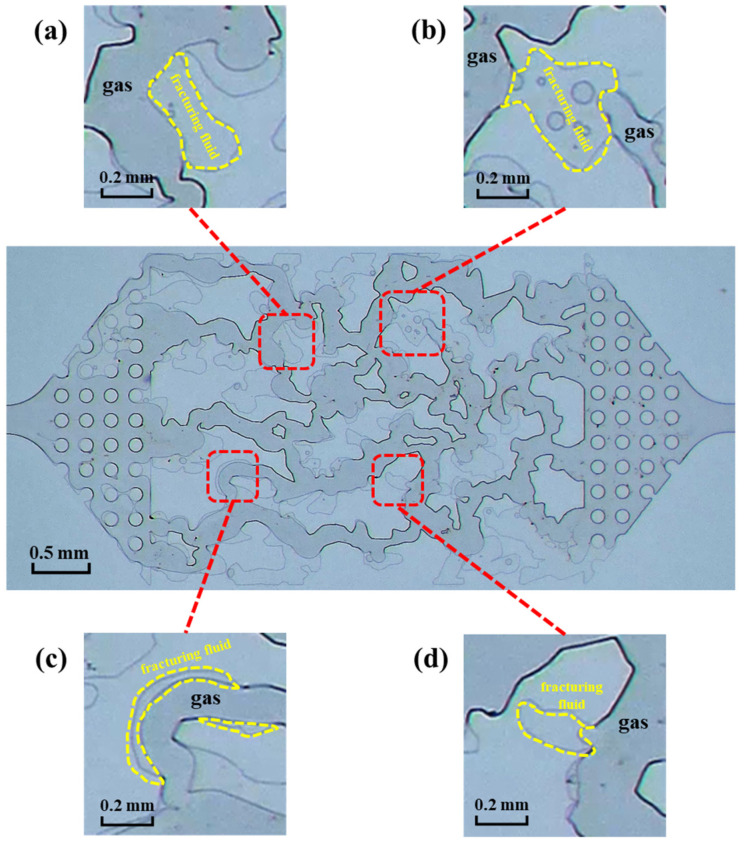
Four kinds of fracturing fluid retention in the microscopic model, fracturing fluid is in the light area, nitrogen is in the dark area and the residual fracturing fluid is marked by the yellow dashed box. (**a**) the viscous retention, (**b**) the Jamin effect, (**c**) the gas blockage effect, (**d**) the dead end of the pore.

Based on the findings of the image analysis, it has been determined that there exist four types of fracturing fluid retention within the microscopic model:

1. Viscous retention occurs when the fracturing fluid adheres to the solid phase surface due to the force of viscosity, as shown in Figure 12a;
2. As the pore radius changes, the fracturing fluid is not easily displaced at the smaller pore throat due to the Jamin effect (The presence of bubbles can retard the flow of water as it progresses through the pore throat of small diameter. The Jamin effect is defined as the resistance to flow under pressure through the smaller pore throat [27]), as shown in Figure 12b;
3. The gas blockage effect (At the same pressure gradient, the gas flows faster than the liquid due to its lower viscosity and the relative permeability of the gas phase is higher in the porous media; once the gas phase controls the flow passages to the main fracture/wellbore, the liquid phase may not be able to flow as it is blocked by the gas) causes the fracturing fluid to be trapped and unable to flow, thus resulting in the fracturing fluid being retained at the pore wall, as shown in Figure 12c;
4. The fracturing fluid becomes confined in the dead end, as shown in Figure 12d.

The main causes of the fracturing fluid retention described above are two types of resistance to the fracturing fluid flowback process: viscous force and capillary force.

#### 4.1.1. Viscous Force

The main mechanism of viscous retention, shown in Figure 12a, is closely related to the viscous force.

**Figure 13:**
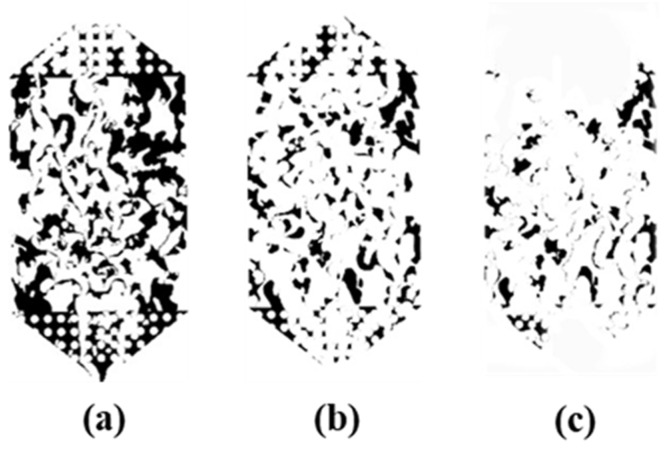
Binary images of the residual fracturing fluid (1.2 wt%) after flowback (**a**) 0.3 MPa gas drive, (**b**) 0.4 MPa gas drive, (**c**) 0.5 MPa gas drive.

Viscosity is a special property of fluids. In a flowing fluid, if the layers are flowing at different velocities, there will be a pair of forces acting and reacting to slow down the original faster layer of flow and speed up the slower one. This pair of equal and opposite forces impede the relative motion of the flow layer. This property of the fluid is called viscosity, and this pair of forces is the viscous force. Viscous retention is an important mechanism of fracturing fluid retention in gas-fracturing fluid two-phase systems. As a polymer solution, PAM fracturing fluid has a reticulated structure. When flowing through porous media at low velocity, the shear effect is weak and the intermolecular forces between polymer molecules are strong, making it difficult to destroy the reticulated structure of the polymer solution [28,29]. As a result, polymer molecules accumulate in the porous media and the retention of fracturing fluid increases. As the gas driving pressure difference increases, the shearing action increases, the reticulated structure of the polymer solution is destroyed and the fracturing fluid retention is significantly reduced, as shown in Figure 13.

According to the Poiseuille formula, the volume of fracturing fluid driven out of the capillary is

(2) $$Q=\frac{\pi r^{4}\left(P_{d}\frac{2\sigma \cos\theta }{r}\right)}{8\mu L}$$

where *r* is the capillary radius, mm; *P_d_* is the driving pressure difference, Pa; *σ* is the gas-fracturing fluid interfacial tension, mN/m; *θ* is the three-phase contact angle of glass/fracturing fluid/gas, °; *μ* is the fracturing fluid viscosity, mPa·s; *L* is the length of fracturing fluid infiltration, mm.

According to Equation (2), viscous retention is mainly controlled by the combination of fracture width, fracture length, driving pressure difference and fracturing fluid viscosity.

**Figure 14:**
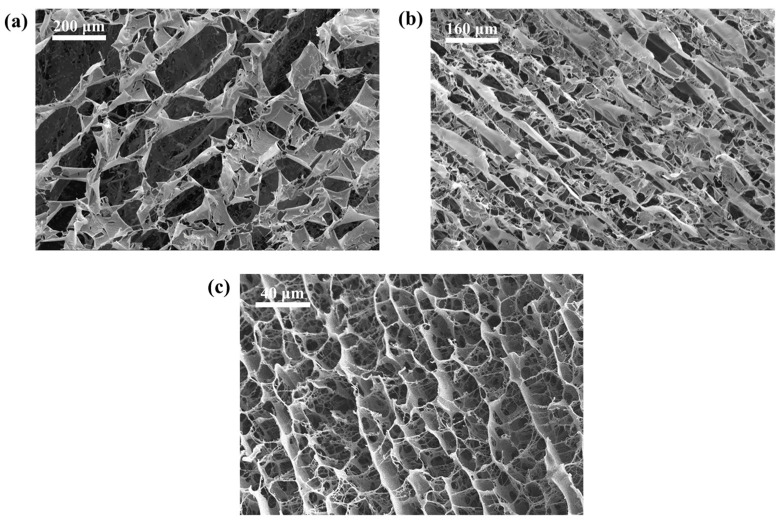
cryo-SEM images of fracturing fluid (**a**) 0.1 wt%, (**b**) 0.8 wt%, (**c**) 1.2 wt%.

As the concentration of the fracturing fluid increases, the microstructure of the polymer solution changes. Experiments were carried out using a HITACHI Regulus 8100 cryogenic scanning electron microscope (cryo-SEM) to observe the microstructure of the fracturing fluid, as shown in Figure 14. The images obtained in the experiment were analyzed using ImageJ software (ImageJ 1.53e)to calculate the average microscopic grid diameter of the fracturing fluid at different concentrations. The results showed that the values corresponding to 0.1 wt%, 0.8 wt% and 1.2 wt% fracturing fluid were 97.8 μm, 55.1 μm and 18.0 μm, respectively.

**Figure 15:**
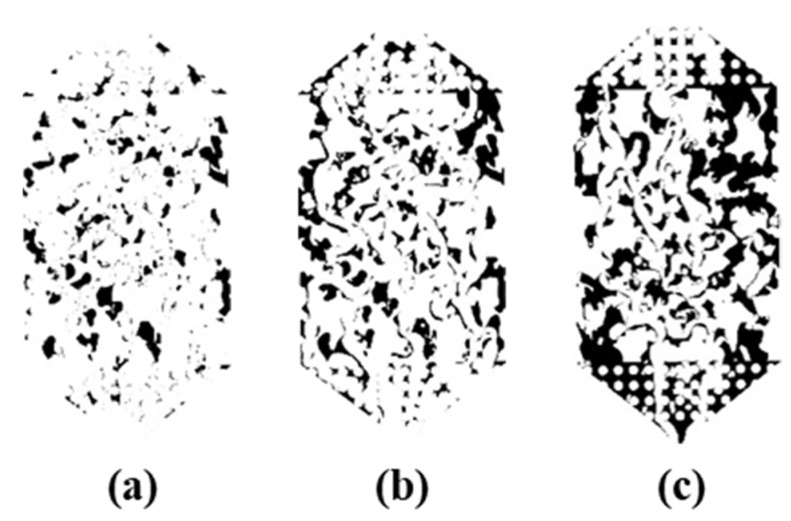
Binary images of the residual fracturing fluid after 0.3 MPa gas drive (**a**) 0.1 wt%, (**b**) 0.8 wt%, (**c**) 1.2 wt%.

The main component of the fracturing fluid is the polymer hydrogel, where the polymer molecules are disordered in the solution, forming a coarse and uneven network structure. As concentration rises, the reticulated structure densifies, heightening intermolecular forces and rendering the structure more resistant to be destroyed during flowback, thereby considerably enhancing fracturing fluid retention. As shown in Figure 15, the residual fracturing fluid volume is positively correlated with the concentration after 0.3 MPa gas drive.

#### 4.1.2. Capillary Force

The main mechanism of fracturing fluid retention caused by the Jamin effect, the gas blockage effect and the dead end of the pore, shown in Figure 12b–d, is closely related to the capillary force. In the two-phase system of fracturing fluid and gas, where the gas is the non-wetting phase, flowback is a process by which the fracturing fluid is displaced. Due to the imbibition under the capillary force, the fracturing fluid would invade into the secondary fracture and matrix. The capillary force is the resistance during the fracturing fluid flowback. The expression of the capillary force in the microscopic model is:

(3) $$p_{c}=\frac{2\sigma \cos\theta }{r}$$

where *σ* is the gas-fracturing fluid interfacial tension, mN/m; *θ* is the three-phase contact angle of glass/fracturing fluid/gas, °; *r* is the capillary radius, mm.

During the gas drive process, the fracturing fluid in the dominant channel is driven out first, with the residual fracturing fluid remaining mainly as a “thin film” on the wall due to low capillary forces. Meanwhile, in the secondary fracture and pore, the residual fracturing fluid remains as a “thick film” with high saturation. Capillary force is the major factor in the retention of residual fracturing fluid both on the wall and in the pore. From Equation (3) it can be seen that the capillary force gradually increases as the pore radius decreases. Shale gas reservoirs contain a large fraction of nano pores [30]. Consequently, the capillary force in shale reservoirs is strong, and a large amount of fracturing fluid would migrate into the matrix.

### 4.2. Flowback Efficiency and Capillary Number

**Figure 16:**
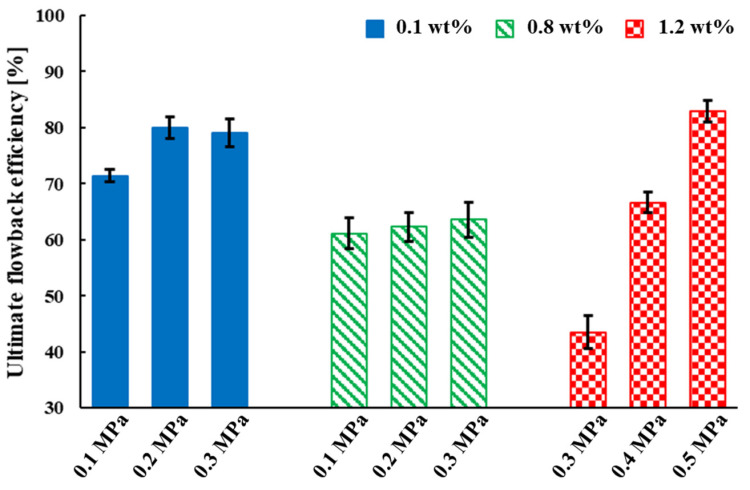
The ultimate flowback efficiencies for nine experimental groups.

For the three fracturing fluid concentrations, a total of nine groups of experiments, as shown in Figure 16, the ultimate fracturing fluid flowback efficiencies showed the following trends:

(1) The ultimate fracturing fluid flowback efficiencies decreased as the fracturing fluid concentration increased;
(2) The ultimate fracturing fluid flowback efficiencies increased as the gas driving pressure differences increased, and this phenomenon was more obvious in the high concentration fracturing fluid.

The fracturing fluid flowback efficiency is influenced by a variety of factors that vary over a wide range, and it is difficult to analyze the complex relationships between these parameters when discussing the effect of a particular factor on fracturing fluid flowback efficiency. The normalization approach is therefore an effective way of analyzing the results of the interaction of these factors. The capillary number, the dimensionless number proposed by Wilkinson [15,31], is widely used in the kinetic analysis of immiscible two-phase displacements [32,33]. The fracturing fluid flowback is theoretically a competition among various forces, which can be characterized by the capillary number [34]. After the formation of the dominant channel, the flowback processes of the fracturing fluid were driven at a constant flow rate. The capillary number is defined by Equation (4).

(4) $$Ca=\frac{v\mu }{\sigma \cos\theta }$$

where *v* is the average flow velocity of the fluids in the microscopic model, m/s; *μ* is the nitrogen viscosity, mPa·s; *σ* is the gas-fracturing fluid interfacial tension, mN/m; *θ* is the three-phase contact angle of glass/fracturing fluid/gas, °.

**Figure 17:**
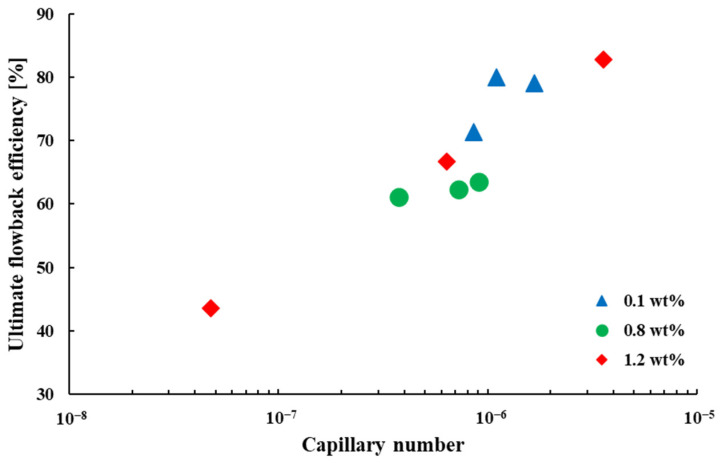
Relationship between the ultimate flowback efficiency of fracturing fluid and capillary number.

The capillary number for each experimental group is given in Appendix A Table A1. The relationship, shown in Figure 17, is similar to the classic two-phase capillary desaturation curve (CDC) for oil and water in low permeability reservoirs. The CDC curve reveals the flow conditions required for oil displacing in porous media [35,36]. As shown in Figure 17, the ultimate flowback efficiency of the fracturing fluid increases with increasing capillary number, and the two also show a high linear positive correlation. In all nine groups of experiments, the capillary numbers were less than 4 × 10^−6^, which are at very low values. In fact, at very low values of capillary number, capillary forces are dominant and the effect of other forces on fracturing fluid retention is modest [37], as can also be seen by Equations (3) and (4). The two-phase capillary desaturation curves obtained from microscopic model experiments are useful for studying the variation pattern of fracturing fluid flowback efficiency.

## 5. Conclusions

In this study, microscopic model experiments are performed to investigate the effects of fracturing fluid concentration and driving pressure difference on fracturing fluid flowback efficiency. The reticulated structure of fracturing fluid polymer molecules is analyzed from a microscopic perspective. The effects of viscous and capillary forces on fracturing fluid retention are discussed and the relationship between capillary number and fracturing fluid flowback efficiency is analyzed. Based on the experimental results, the following conclusions can be drawn.

(1) In the early stages of fracturing fluid flowback, flowback efficiency increases rapidly and then plateaus. The ultimate fracturing fluid flowback efficiencies decreased as the concentration increased or the gas driving pressure differences decreased.
(2) There are four kinds of mechanisms responsible for fracturing fluid retention in the pore network: viscous resistance, the Jamin effect, the gas blockage effect and the dead end of the pore. The viscous force and the capillary force are two important resistances to the fracturing fluid flowback process.
(3) The ultimate flowback efficiency of the fracturing fluid increases linearly with increasing capillary number. At very low capillary numbers, capillary forces dominate fracturing fluid retention.

## Figures and Tables

**Table 1:** The function profile of polymers used in water-based fracturing fluids.

Polymer	Functional Profile
Polyacrylamide (PAM)	increases viscosity and thickness to enhance hydraulic effect and reduce fluid loss
Hydroxyethyl Cellulose (HEC)	increases viscosity and gel properties to prevent fluid loss and enhance fracture formation
Cellulose	increases viscosity and gel properties to improve rheological properties and shear resistance
Polyamide Gel (PAG)	prevent fluid loss and enhance fracture stability
Polyvinyl Alcohol (PVA)	increases viscosity and gel properties to prevent fluid loss and enhance fracture formation

**Table 2:** The differences between water-based and silicate fracturing fluids.

Comparison Item	Water-Based Fracturing Fluid	Silicate-Based Fracturing Fluid
Composition	Water and some additives, such as viscosity agents, dispersants, emulsifiers, etc.	Chemicals such as water glass, silicates, phosphates, etc.
Application Range	Suitable for softer and more brittle formations, such as shale gas, shale oil, tight oil, etc.	Suitable for harder and more dense formations, such as sandstone, conglomerate, limestone, etc.
Fracturing Effect	Forms certain porosity to increase rock permeability during the fracturing process	Generates chemical reactions with formation minerals to produce gels, increasing formation strength and stability
Environmental Friendliness	Relatively more environmentally friendly, with lower risk of groundwater and environmental pollution	Has a greater impact on the environment because it contains chemicals

**Table 3:** The properties of the fluids.

Concentration (wt%)	Viscosity (mPa·s)	Interfacial Tension (mN·m^−1^)	Contact Angle (°)
0.1	7.1	54.5	35.02
0.8	62.7	72.9	38.49
1.2	96.1	87.7	50.50

**Table 4:** Summary of experimental results.

Concentration (wt%)	Viscosity (mPa·s)	Gas Driving Pressure Difference (MPa)	*S_f_* (%)	*FB* (%)
0.1	7.1	0.1	28.58	71.42
		0.2	20.01	79.99
		0.3	20.89	79.11
0.8	62.7	0.1	38.84	61.16
		0.2	37.69	62.31
		0.3	36.43	63.57
1.2	96.1	0.3	56.48	43.52
		0.4	33.34	66.66
		0.5	17.14	82.86

## Data Availability

Not applicable.

## Supplementary Materials

The following supporting information can be downloaded at: https://www.mdpi.com/article/10.3390/polym15061560/s1, Videos S1–S9: The process of fracturing fluid flowback in the microscopic model.

## Appendix A

**Table A1:** List of the main parameters.

Concentration (wt%)	Interfacial Tension (mN·m^−1^)	Contact Angle (°)	Flow Rate (m/s)	Capillary Number
0.1	54.5	35.02	2.185 × 10^−3^	8.6008 × 10^−7^
			2.790 × 10^−3^	1.0983 × 10^−6^
			4.232 × 10^−3^	1.6660 × 10^−6^
0.8	72.9	38.49	1.219 × 10^−3^	3.7534 × 10^−7^
			2.351 × 10^−3^	7.2407 × 10^−7^
			2.934 × 10^−3^	9.0370 × 10^−7^
1.2	87.7	50.50	1.510 × 10^−4^	4.7528 × 10^−8^
			2.028 × 10^−3^	6.3876 × 10^−7^
			1.135 × 10^−2^	3.5754 × 10^−6^
